# The Effects of Omega 3 and Omega 6 Fatty Acids on Glucose Metabolism: An Updated Review

**DOI:** 10.3390/nu15122672

**Published:** 2023-06-08

**Authors:** Filippo Egalini, Ornella Guardamagna, Giulia Gaggero, Emanuele Varaldo, Beatrice Giannone, Guglielmo Beccuti, Andrea Benso, Fabio Broglio

**Affiliations:** 1 Division of Endocrinology, Diabetes and Metabolism, Department of Medical Sciences, University of Turin, 10126 Turin, Italyguglielmo.beccuti@unito.it (G.B.);; 2 Paediatric Endocrinology, Department of Public Health and Paediatric Sciences, University of Turin, 10126 Turin, Italy

**Keywords:** eicosapentaenoic acid, docosahexanoic acid, arachidonic acid glucose metabolism disorders

## Abstract

Massive changes have occurred in our diet. A growing consumption of vegetal oils rich in omega-6 (ω-6) and a depletion of omega-3 (ω-3) fatty acids (FAs) in our food has led to an imbalance between ω-3 and ω-6. In particular, eicosapentaenoic (EPA)/arachidonic acid (AA) ratio seems to be an indicator of this derangement, whose reduction is associated to the development of metabolic diseases, such as diabetes mellitus. Our aim was therefore to investigate the literature on the effects of ω-3 and ω-6 FAs on glucose metabolism. We discussed emerging evidence from pre-clinical studies and from clinical trials. Notably, conflicting results emerged. Source of ω-3, sample size, ethnicity, study duration and food cooking method may be responsible for the lack of univocal results. High EPA/AA ratio seems to be a promising indicator of better glycemic control and reduced inflammation. On the other hand, linoleic acid (LA) appears to be also associated to a minor incidence of type 2 diabetes mellitus, although it is still not clear if the outcome is related to a reduced production of AA or to its intrinsic effect. More data derived from multicenter, prospective randomized clinical trials are needed.

## 1. Background

Omega-3 (ω-3) and omega-6 (ω-6) fatty acids (FAs) are two classes of essential dietary polyunsaturated fatty acids (PUFAs) derived, respectively, from α-linolenic acid (ALA, 18:3 ω-3) and linoleic acid (LA, 18:2 ω-6) [1]. The distinction between ω-3 and ω-6 FAs is based on the location of the first double bond, counting from the methyl end of the FA molecule: the first double bond is between the 3rd and 4th carbon atom for the ω-3 FAs, while it is between the 6th and 7th carbon atom in the ω-6 FAs [2]. Such PUFAs, because of the lack of endogenous enzymes for ω-3 desaturation, cannot be synthetized by humans and by other mammals and therefore must be derived from the diet. LA is present in a large number of foods in nature and is found in particular in the seeds of most plants; exceptions are cocoa, palm and coconut. ALA, conversely, is less common: it is contained in the chloroplasts of green leafy vegetables and in walnuts, seeds of chia, flax, perilla and rape [3]. Other important sources of ALA are hemp seed oil and soybean oil (ALA content about 17% and 8%, respectively) [4,5]. However, despite various easily accessible soybean-derived products have an unchanged content of FAs (e.g., tempeh, tofu, etc.), others have a reduced content of FAs (e.g., soy milk, textured vegetable proteins, etc.) compared to the original source [6,7]. Foods and oils rich in ALA and LA are displayed in Table 1.

**Figure 1:**
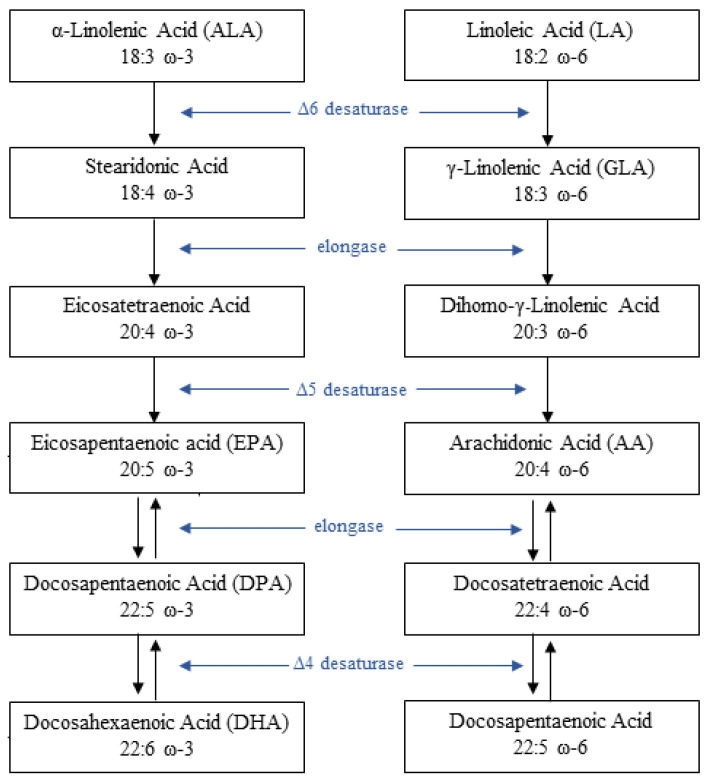
Metabolism of α-linolenic acid (ALA) and linoleic acid (LA).

Once ingested, both LA and ALA are subsequently metabolized to longer-chain FAs of 20 and 22 carbon atoms by increasing the chain length and the degree of unsaturation of the FA molecule (Figure 1) [8]. LA can be converted to arachidonic acid (AA, 20:4 ω-6) while fate of ALA is the transformation into eicosapentaenoic acid (EPA, 20:5 ω-3) and docosahexaenoic acid (DHA, 22:6 ω-3).

Anyway, such a conversion process is slow, [9,10,11], especially in men [12], and both ω-3 and ω-6 FAs compete for the same desaturation enzymes (mainly Δ5 and Δ6 desaturase, respectively also known as Fatty Acid Desaturase 1-2, FADS 1-2) [2]. Moreover, the conversion of ALA into EPA and DHA is decreased when the amount of LA in the diet increases [11]. For such a reason, both EPA, DHA and AA can be assumed directly with the diet. The latter is found typically in the phospholipids of eggs, dairy and grain-fed animals, whereas the former are mainly contained in fatty fish, also known as “oily fish”. Furthermore, nowadays several supplements of ω-3 PUFAs are available: fish oils, cod liver oil, krill oil and some algal oils, and eventually concentrated pharmaceutical-grade preparations of EPA and DHA, or EPA alone [13,14].

However, ω-6 and ω-3 FAs are metabolically and functionally distinct; moreover, they are not interconvertible and often have markedly opposite physiological effects. Although observational studies provided evidence that LA plays a protective role in cardiovascular health, it can still be converted to AA that can have negative effects [15]. Indeed, AA metabolism can produce eicosanoids such as prostaglandin (PG) E2 and leukotriene (LT) B4, which are mediator molecules of thrombosis and inflammation in a more marked way than similar products derived from ω-3 PUFAs (PGE3 and LTB5 synthesized from EPA) [2,16,17]. However, oxylipins with an anti-inflammatory effect can be synthetized as well [18]. Eventually, from the enzymatic processing of EPA and DHA anti-inflammatory mediators (e.g., resolvins, maresins and protectins) are produced [19].

This considered, it appears evident how the ω-6 and ω-3 balance in the diet is extremely important [2].

Over the last decades, great changes have taken place in the food habits of the Western countries. As of today, the modern diet is based on greater consumption of vegetal oils rich in ω-6 FAs and foods rich in trans FAs with a consequent ω-3 FAs reduced intake [2]. In fact, the massive adoption of intensive breeding in recent times has led to a significant reduction in the ω-3 content in many products including eggs, animal meats and even fish [2,20,21,22].

The reduced content of ω-3 in fish has become an important issue. Nowadays fish is one of the major sources of ω-3 FAs in humans and, as a result, an increase of its consumption is considered to be a way to contrast the above-mentioned imbalance between ω-6 and ω-3. However, Van Vliet et al. already showed back in 1990 that farmed fish contains less ω-3 FAs than wild fish caught in the ocean, rivers and lakes [22]. More recently, Sprague et al. comparing the FAs composition of farmed fish from 2006 to 2015, found that EPA and DHA content significantly decreased over the years [23]. In fact, microalgae and phytoplankton are the real sources of EPA and DHA in aquatic ecosystems and today the died of farmed fish lacks them, as it consists of grains, plants and fishmeal [24]. Moreover, intensive fish farming poses a high environmental risk due to the feeding needs and the chemicals used associated with the production process [25]. Modern commercial fishing is not the solution as well, since it causes a significant depletion of plankton and, overall, has a massive detrimental impact on marine ecosystems [26,27]. As a result, some authors advocate the adoption of microalgae oil and plant-based seafood analogs as sustainable sources of EPA and DHA [28,29,30].

In the same way, the ω6/ω3 ratio is significantly altered in poultry and egg yolk deriving from breeding [21]. Finally, exploitation of cultivated fields has led to an increase in such ratio with respect to wild plants [31]. As a result, today’s Western diet can be considered ω3-deficient with an unbalanced ω6/ω3 ratio. This ratio, nowadays, varies according to the populations and geographical areas considered, from a minimum of 15–20 up to a maximum of 40–50 [2,3] and is much higher than that of wild animals [32,33,34,35,36].

Interest in ω-3 FAs (and in particular EPA) has increased exponentially especially in recent years, although a possible beneficial effect on health has been known at least since the 1970s [37]. Indeed, there is evidence to support that high consumption of wild fish (as is customary in some populations such as Greenland Eskimos, Icelanders, Inuit indigenous and Native Americans in Alaska) is associated to low rates of atherosclerotic disease, autoimmune disorders, obesity and cancer [2,38,39,40,41,42].

Accordingly, recent guidelines on nutrition suggest an increase in the human diet of ω-3 PUFAs rich foods [43,44]. In particular, daily recommended intake for ω-3 PUFAs varies worldwide and for different age groups: it usually ranges between 250 and 500 mg/day (the equivalent of at least 2 portions of fish a week) and it is higher in infants and in pregnant and lactating women [11,45,46,47,48]. However, experts estimated that less than 20% of the world’s human population consumes ≥250 mg/day of ω-3 PUFAs [7].

Recent cardiovascular guidelines, in addition, suggest prescribing ω-3 PUFAs supplements in a subgroup of hypertriglyceridemic patients at high cardiovascular risk [49]. However, some authors call for caution highlighting the lack of decisive evidence on ω-3 PUFAs supplementation [50]. 

The skepticism toward this topic is because past clinical trials on ω-3 PUFAs supplementation such as VITAL, GISSI-HF and ASCEND, could not show any effect of 1 g/day of combinations of EPA and DHA on cardiovascular events [51,52,53].

Recent trials such as JELIS, REDUCE-IT, EVAPORATE and RESPECT-EPA, though, found a striking reduction in cardiovascular events in high-cardiovascular risk patients treated with supplement of EPA (or IPA, a highly purified and stable EPA ethyl ester) [54,55,56,57]. In agreement with the suggested switch to an EPA-only approach, a similar study, named STRENGHT Trial, failed to prove a reduction in cardiovascular events in an analogue population treated with a combination of EPA and DHA, 4 g/day (with an EPA/DHA ratio of 1,2) [58]. 

Based on this new evidence, the 2019 European Society of Cardiology (ESC) and European Atherosclerosis Society (EAS) guidelines for dyslipidemia proposed to consider the prescription of EPA 4 g/day in subjects with established cardiovascular disease with triglycerides (TGs) between 150 to 499 mg/dL despite statin treatment, versus the previously common use of 1–2 g/day of the combination EPA + DHA [59]. This approach was later confirmed by the 2021 ESC guidelines focused on Cardiovascular Prevention [49].

The reason for this effect has not been fully elucidated yet, although researchers think that the anti-inflammatory effect of EPA may have played a crucial role [60,61].

In this spirit, it is understandable why some researchers began to investigate deeply the ratio ω3/ω6, and in particular EPA/AA. As previously stated, AA is a precursor to a number of pro-inflammatory/pro-aggregatory mediators. Conversely, EPA acts competitively with AA for the key cyclooxygenase and lipoxygenase enzymes and, as a result, it exerts an anti-inflammatory activity [62]. It also appears that EPA has relevant anti-oxidant effect, that seems to be minimal or absent in DHA [63,64].

Scientists demonstrated that treatment with ω-3 PUFAs supplements in different combinations is associated with an augmented EPA/AA ratio [65,66,67,68]. Moreover, not only supplementation, but also a diet rich in ω-3 PUFAs (both plant-based and fish-derived food) can enhance EPA/AA ratio [69,70,71].

In accordance to what expected, experimental studies showed that a higher EPA/AA ratio is associated with reduced inflammation (reduction in C reactive protein) and arterial stiffness [72]. Subsequently, researchers outlined that an increase of this ratio reduces cardiovascular risk and that its reduction is linked with metabolic dysfunctions like non-alcoholic fatty liver disease (NAFLD) and obesity [3,54,73,74,75].

As previously stated, population groups whose diet is rich in oily fish, have a lower prevalence of chronic diseases, including diabetes mellitus (DM) and impaired fasting glucose (IFG) [41,76]. As a result, EPA/AA ratio was adopted as a parameter in various studies to evaluate the possible beneficial effect of this parameter on glucose metabolism [65,68].

The aim of this review is therefore to investigate recent literature to try to unravel this possible additional outcome of ω-3 PUFAs on glucose metabolism.

## 2. Pathophysiology of PUFAs and Glucose Metabolism Interaction

The incidence of type 2 diabetes mellitus (T2DM) has significantly increased during the last 30 years, with an estimated rise of 102.9% worldwide [77]. Several aspects are implicated (e.g., aging, lack of physical activity, smoking etc.), but diet seems to be a major cause, both in terms of higher volume and poorer quality of food [78]. An excessive intake of sugars, for instance, enhances the risk of developing T2DM [79].

Moreover, a further element could be the large use of vegetable oils rich in ω-6 FAs and the reduced consumption of ω-3 FAs rich foods. In fact, AA induces a pro-inflammatory state, which represents a fertile ground for the development of insulin resistance (IR), especially via a process interleukin-1β (IL-1 β) and tumor necrosis factor-α (TNF-α) dependent [80].

Another factor is the growing use of refined carbohydrates (CHO), which represent both a stressful stimulus for pancreatic cells and a catalyst of AA production. In fact, the rapid insulin peak that follows a meal rich in refined CHO, promotes the biochemical cascade from LA to AA, activating the enzymes Δ5 and Δ6 desaturase [3].

**Figure 2:**
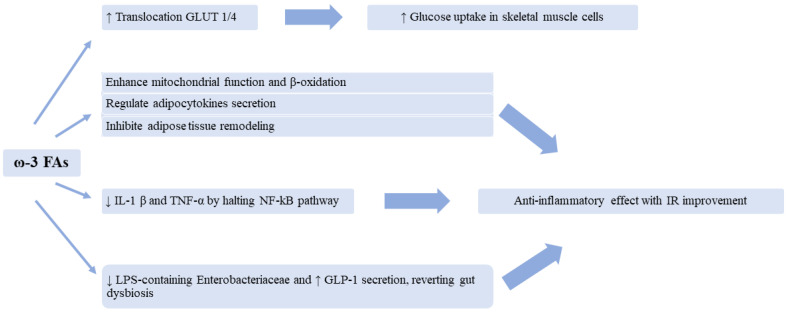
Summary of the pathophysiological mechanisms of Omega-3 Fatty Acids (ω-3 FAs) on glucose metabolism. ↑: increased, ↓: reduced, GLUT1/4: GLUcose Transporter 1/4, IL-1 β: Interleukin-1β, TNF-α: Tumor necrosis factor-α, LPS: Lipopolysaccharide, GLP-1: Glucagon-like peptide 1, IR: Insulin resistance.

On the other hand, ω-3 FAs seem to have a significantly positive impact on glucose metabolism, as emerged from epidemiological studies of populations groups whose diet rich in fish, as above-mentioned (Figure 2) [41,76]. Indeed, experts demonstrated in vitro that a treatment with EPA leads to the promotion of glucose uptake in human skeletal muscle cells. This process seems to be due to both an augmented translocation of GLUT1/4 (GLUcose Transporter 1/4) to the plasma membrane and to GLUT-independent mechanisms [81,82,83].

Moreover, ω-3 FAs are also able to ameliorate IR probably enhancing mitochondrial function and β-oxidation, regulating adipocytokines secretion and inhibiting adipose tissue remodeling [84,85,86,87,88,89]. 

Among the positive metabolic outcomes of ω-3 FAs, the anti-inflammatory effect stands out. EPA and DHA, indeed, halt NF-kB pathway leading to reduced levels of IL-1 β and TNF- α, cytokines implicated in the development of IR [90,91,92].

Finally, an intriguing aspect is the possibility of ω-3 FAs of reverting gut dysbiosis, caused by a diet rich in saturated fat [93,94,95]. This is a pro-inflammatory condition that increases the influx of lipopolysaccharide (LPS) which further triggers systemic inflammation and IR by activating toll-like receptor 4 (TLR4) [96]. Scientists demonstrated that a treatment with ω-3 FAs, and especially EPA, attenuates hyperglycemia and IR in diabetic mice, while having no significant impact on body weight. Among all the recorded effects, the supplementation reduced the abundance of the LPS-containing Enterobacteriaceae and promoted glucagon-like peptide 1 (GLP-1) secretion [97].

## 3. Evidence from the Literature

Considering the important underlying pathophysiological rationale, over the last years researchers have conducted studies to understand whether blood PUFAs levels can correlate or even predict the risk of developing metabolic disorders.

Several meta-analyses investigated the role of LA on the development of T2DM. The results outlined that high intake of dietary LA and elevated concentrations of LA in the body were both significantly associated with a lower risk of T2DM, suggesting a possible protective factor of this FA [98,99]. Although these reports do not have a clear explanation yet, scientists hypothesized that LA and its metabolites act as agonists of peroxisome proliferator-activated receptors (PPARs) with net insulin-sensitizing, lipid-lowering and anti-inflammatory effects [100]. It should be noted, however, that high levels of LA may be the result of both a high dietary intake but also a possible low activity of Δ5 and Δ6 desaturase because of genetic variants of these enzymes, leaving uncertainty on the matter [101]. Indeed, some authors specify that LA might be metabolically harmful when converted to AA, precursor of the inflammatory cascade [98].

This observation may lead to a more cautious interpretation of the beneficial cardiometabolic outcomes of LA.

Similarly, large, prospective studies and meta-analysis could not found univocal associations between ω-3 FAs and T2DM [99,102,103,104,105,106,107,108,109]. A possible explanation may be the great heterogeneity between the included studies, mostly regarding the source of ω-3 (e.g., farmed versus wild fish), sample size, different ethnicity, study duration and, not least, food cooking method.

Self-reported, although validated, food frequency questionnaires may lack major information regarding the cooking method, whose importance is becoming more and more evident since several studies have already showed how this could compromise the positive effects of ω-3 on health [110,111].

In fact, as in many countries fish is cooked, rather than eaten raw, researchers focused their attention on the possible alteration of ω-3 FAs during the cooking process.

Some authors stated that when fish is exposed to high temperatures, ω-3 FAs content can be reduced, whereas others found that if fish is pan-fried, for instance, EPA and DHA can be modified and the process can even generate detrimental bioactive compounds called oxidized PUFA products [110,111,112]. To overcome these issues, it would be preferable to focus on studies based on EPA or EPA + DHA supplementation, in order to have a known ω-3 dose. Moreover, even better may be to perform a blood analysis of EPA and DHA concentration.

Consequently, several studies were conducted over the years to evaluate the effects of ω-3 FAs supplementation on glucose metabolism.

In 2021, Al Rijjal et al., used two highly pure ω-3 ethyl ester prescription drugs, Icosapent Ethyl (IPE, a purified ester of EPA, dosage 1 g/day) and a combination of EPA + DHA (respectively 465 mg and 375 mg), to treat mice for one week prior to six weeks of high-fat diet (HFD) feeding [113]. EPA + DHA was given at an equivalent of 2 g/day in humans so that the EPA content was the same compared with IPE. The researchers found that mice treated with IPE had significantly lower weight compared to control-HFD mice. IPE-treated mice also had significantly reduced fasting blood glucose and insulin, reduced insulin secretion (apparently through an improvement in the homeostasis model assessment for IR [HOMA-IR] and insulin sensitivity) and had improved glucose tolerance. In comparison, the effects of mice treated with EPA + DHA were less pronounced in all the outcomes analyzed. Interestingly, improvements in glucose homeostasis with either IPE or EPA + DHA treatment were only apparent under conditions of HFD. The authors suggested that these effects might be due to the upregulation of FA oxidation leading to reduced hepatic TGs, changed metabolism of branched-chain amino acid (BCAA) and sphingolipids and mitigated inflammation.

Similarly, Sawada et al. attempted to investigate the effects of 6-month EPA treatment on postprandial hyperglycemia and hyperlipidemia, insulin secretion, and concomitant endothelial dysfunction in patients with IFG but with no established diagnosis of T2DM. The authors subjected these individuals to oral glucose tolerance test (OGTT). Patients with impaired glucose tolerance (IGT, blood glucose after 2 h between 140 and 199 mg/dL) and those with a new diagnosis of DM (blood glucose after 2 h ≥ 200 mg/dL) were classified as subjects with impaired glucose metabolism (IGM). They randomized patients with IGM and CAD (coronary artery disease) to receive either 1800 mg/day of EPA or no treatment. After six months, the EPA group showed, in addition to an increase in the EPA/AA ratio, a significant benefit of the lipid profile and an improvement in glucose homeostasis in terms of both postprandial blood glucose and insulin secretion [114]. These results thus seem to support that EPA might provide a greater protective effect against developing TD2M among pre-diabetic patients.

Recently, it has been suggested to switch the focus from the absolute to the relative increase of ω-6 FAs over ω-3 FAs [3]. Indeed, an unbalanced ratio not only promotes a prothrombotic and proinflammatory state, but also increases IR, which can lead to both obesity and diabetes. In particular, a correlation was observed between a lower EPA/AA ratio and a greater prevalence of silent inflammation, a risk factor for metabolic diseases [3]. 

**Figure 3:**
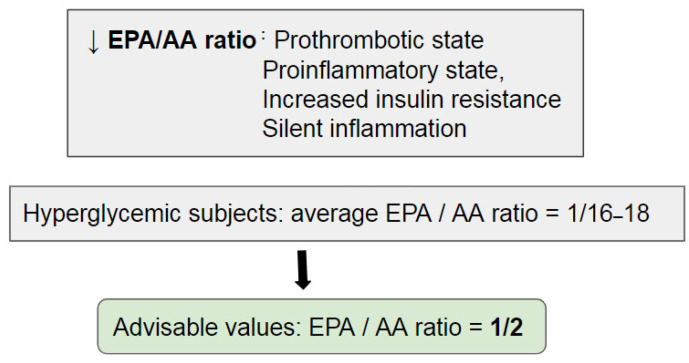
Detrimental effects of low EPA/AA ratio, average hyperglycemic values and advisable values [116]. EPA: Eicosapentaenoic Acid, AA: Arachidonic Acid, ↓: reduced.

Indeed, hyperglycemic subjects have an average EPA/AA ratio of 1:16–18, while a value less than 1:2 should be advisable (Figure 3) [115].

Of note, a recent trial highlighted that the maternal EPA/AA ratio could also be used to search for women affected by gestational diabetes who are at higher risk of needing drug treatment [116].

However, a small retrospective study published in 2023 on Arabic patients revealed a higher prevalence of an elevated EPA/AA ratio but also an augmented ω6/ω3 ratio among hyperglycemic patients, confirming the lack of decisive results on the role of PUFAs in the glucose homeostasis [117].

Recent studies focused also on patients with a known diagnosis of T2DM. As expected, these patients were found to have a disproportionate ratio in favor of ω-6 [115].

An observational study on 1733 Japanese diabetic individuals found that a lower level of EPA/AA is associated to higher systemic inflammation and to prior myocardial infarction [118]. In addition, the authors pointed out that the above-cited ratio, unlike DHA/AA ratio, is not affected by statin use, a class of drugs commonly prescribed to these patients.

Another study on diabetic subjects with atherosclerotic cardiovascular disease showed that participants with a glycated hemoglobin ≥7% had a lower EPA/AA ratio and a higher ω6/ω3 ratio [119].

Finally, the ratio has been studied even in a type 1 diabetes mellitus (T1DM) pediatric population. Results show that a higher EPA/AA ratio is associated to lower insulin demand [120]. Even though the study involved only 40 participants, this report has relevant implications. In fact, the ratio seems to be involved in glucose metabolism even in a completely different condition, such as T1DM, which has an autoimmune etiopathogenesis. Moreover, confounding factors like obesity, smoking, comorbidities, polypharmacotherapy are usually less common in a pediatric population.

**Figure 4:**
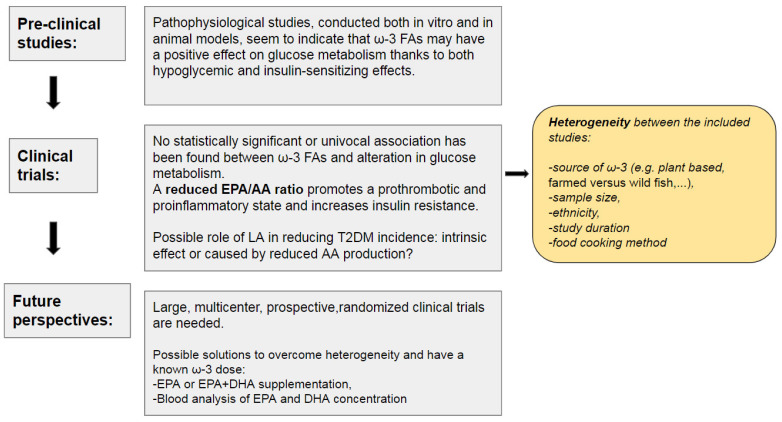
Summary of the evidence from pre-clinical, clinical studies and future perspectives. ω-3 FAs: Omega-3 Fatty Acids, EPA: Eicosapentaenoic Acid, AA: Arachidonic Acid, LA: Linoleic Acid, T2DM: Type 2 Diabetes Mellitus, DHA: docosahexaenoic acid.

Evidence from preclinical, clinical studies and future perspectives is summarized in Figure 4.

## 4. Conclusions

The imbalance between ω-3 and ω-6 FAs in the modern Western diet seems to be a contributor of the marked increase in the incidence of metabolic diseases, such as DM, in the last 30 years. The consequent solution may be to invert the trend in order to obtain a balanced ω3/ω6 ratio. The strategies include either a dietary change, consisting in a switch to a diet rich in ω-3 FAs and low in ω-6 FAs, or a ω-3 oral pharmaceutical supplementation. 

Pre-clinical studies seem to indicate that ω-3 may have a positive effect on glucose metabolism thanks to both hypoglycemic and insulin-sensitizing effects. However, results from clinical trials are inconclusive due to the heterogeneity of the included studies. Source of ω-3, sample size, ethnicity, study duration and food cooking method may be responsible for the lack of univocal results. On the one hand, various evidences show an association between high values of LA and reduced risk of T2DM, although it is still not clear if the outcome is related to a reduced production of AA or to LA intrinsic effect.

On the other hand, low EPA/AA ratio may be a promising indicator of worse glycometabolic control and higher inflammation in both diabetic and non-diabetic patients. Multicenter, prospective randomized clinical trials are needed to gain further understanding on the relation between EPA/AA ratio and glucose metabolism.

## Figures and Tables

**Table 1:** Foods and oils rich in α-linolenic acid (ALA) and linoleic acid (LA), expressed in content (g) per 100 g [5,6].

	ALA (18:3 ω-3)	LA (18:2 ω-6)
Chia seeds	17.8	5.9
Flaxseeds	19.4	5.3
Hemp seeds	8.7	27.5
Walnuts	9.1	38.1
Soybean oil	6.8	51
Corn oil	1.0	51.9
Sunflower oil	0.2	20.5
Avocado oil	0.9	12.5
Extra virgin olive oil	0.7	8.4
Peanut oil	0.3	19.6

## Data Availability

Data sharing not applicable.
